# Theranostic nanoplatforms of emodin-chitosan with blue laser light on enhancing the anti-biofilm activity of photodynamic therapy against *Streptococcus mutans* biofilms on the enamel surface

**DOI:** 10.1186/s12866-022-02481-6

**Published:** 2022-03-04

**Authors:** Maryam Pourhajibagher, Nasrin Keshavarz Valian, Abbas Bahador

**Affiliations:** 1 Dental Research Center, Dentistry Research Institute, Tehran University of Medical Sciences, Tehran, Iran; 2 Department of Periodontics, School of Dentistry, Shahid Beheshti University of Medical Sciences, Tehran, Iran; 3 Fellowship in Clinical Laboratory Sciences, BioHealth Lab, Tehran, Iran

**Keywords:** Antimicrobial photodynamic therapy, Biofilms, Chitosan, Emodin, *Streptococcus mutans*

## Abstract

**Background:**

Combining photosensitizer and light irradiation, named antimicrobial photodynamic therapy (aPDT) is an adjuvant therapy for eliminating microbial biofilms. This ex vivo study evaluates the effect of anti-biofilm activity of aPDT based on emodin-chitosan nanoparticles (Emo-CS-NPs) plus blue laser light against *Streptococcus mutans* biofilm on the enamel surface.

**Materials:**

After determination of the fractional inhibitory concentration index of Emo and CS by checkerboard array assay, Emo-CS-NPs were synthesized and characterized. Following treatment of pre-formed *S. mutans* biofilms on the enamel slabs, cellular uptake of Emo-CS-NPs and intracellular reactive oxygen species (ROS) production were determined. The anti-biofilm and anti-metabolic activities of aPDT were investigated. Eventually, lactic acid production capacity, concentrations of *S. mutans* extracellular DNA (eDNA) levels, and expression of the gene involved in the biofilm formation (*gtfB*) were evaluated.

**Results:**

The maximum uptake of Emo-CS-NPs occurs in an incubation time of 5 min. When irradiated, Emo-CS-NPs were photoactivated, generating ROS, and led to a decrease in the cell viability and metabolic activity of *S. mutans* significantly (*P* < 0.05). *S. mutans* eDNA and lactic acid production outcomes indicated that Emo-CS-NPs-mediated aPDT led to a significant reduction of eDNA levels (48%) and lactic acid production (72.4%) compared to the control group (*P* < 0.05). In addition, *gtfB* mRNA expression in *S. mutans* was downregulated (7.8-fold) after aPDT in comparison with the control group (*P* < 0.05).

**Conclusions:**

Our data support that, aPDT using Emo-CS-NPs revealed the highest cellular uptake and ROS generation. Emo-CS-NPs based aPDT could inhibit significantly biofilm formation and reduce effectively virulence potency of *S. mutans*; thus, it could be an adjuvant therapy against dental caries.

## Introduction

Dental caries is one of the most common diseases in human societies that leads to pain, dental infection, and eventually tooth loss [1, 2]. Dental caries is a multifactorial disease and requires four simultaneous conditions for caries to develop: a prone tooth or host, sufficient caries-causing microorganisms, frequent consumption of large amounts of fermentable carbohydrates, and the passage of time. Dental caries are caused by organic acids as a by-product of bacterial metabolism of fermentable carbohydrates diffusing into enamel and dissolving the mineral [3].

*Streptococcus mutans* is one of the bacteria that play an effective role in dental caries. *S. mutans* as a Gram-positive and optional anaerobic coccus is a microbial component of the oral cavity in humans, whose cariogenic properties have been proven in various studies [2–5]. According to studies, the binding and colonization of *S. mutans* around composites and dental restorations are more than other areas in the mouth. By metabolizing sucrose, S*. mutans* is able to make insoluble extracellular polysaccharides that increase the ability of bacteria to attach to teeth and form biofilms [4]. The stronger adherence is mediated by glucosyltransferases (Gtfs), especially GtfB and GtfC, and glucanbinding proteins (Gbps) [5].

As biofilm formation progresses, microbial species can produce and encase themselves within the extracellular polymeric substance comprised a network of molecules such as polysaccharides, lipids, proteins, and extracellular DNA (eDNA) [6]. The studies focusing on *S. mutans* confirm the role of eDNA in maintaining biofilm structural integrity and stability [7, 8], consistent with findings for other bacterial species [9–11].

There are various methods to inhibit the progress and evolution of oral microbiota which can impair the metabolism of bacteria as well as prevent the formation of dental plaque [12]. Although it is possible to use antibacterial agents for disinfection, it has shown that the use of these agent may increase the resistant species of caries-causing bacteria [13]. Therefore, the development of antimicrobial-resistant species has led to extensive research to find alternative antibacterial therapies.

Antimicrobial photodynamic therapy (aPDT) involves the use of the non-toxic light-sensitive dyes (photosensitizer) in combination with visible light in the presence of oxygen. The light at a certain wavelength can excite and activate the photosensitizer [14]. The excited photosensitizer transfers energy or electrons to oxygen molecules that produce reactive oxygen species (ROS) such as single oxygen or hydroxyl radicals, which leads to the killing of microbial cells [15].

Emodin (Emo; 3-methyl-1,6,8-trihydroxyanthraquinone), as a photosensitizer extracted from plants such as *Rheum palmatum* and *Polygonum cuspidatum*, is a Food and Drug Administration (FDA) approved natural compound. The pharmacological data indicate that Emo has a wide range of biological activities such as antibacterial, antiviral, and anti-cancer effects and its effect on microbial anti-biofilm activity has been reported [16, 17]. Chitosan (CS), sometimes referred to as chitin deacetylated, is a natural linear polycationic polysaccharide derived from the deacetylation of part of chitin [18]. CS is very noteworthy due to its non-toxicity, high adsorption properties, degradability in nature, environmental friendliness, cost-effectiveness, fast synthetics, and finally the possibility of preparing many derivatives from [19]. CS is insoluble at neutral and alkaline pH, but forms water-soluble salts with wide spectrum of inorganic and organic acids [20]. Also, the antimicrobial properties of CS have been reported in many studies [21–24].

According to the literature, there is no study that has shown the synergistic anti-biofilm effects of emodin-chitosan nanoparticles (Emo-CS-NPs) as a new photosensitizer in aPDT. Therefore, the aim of this study was to investigate the anti-biofilm effects of Emo-CS-NPs based aPDT against *S. mutans* biofilm on the enamel surface ex vivo and to determine the in vitro metabolic activity, eDNA level, and intracellular ROS production in *S. mutans* following Emo-CS-NPs based aPDT treatment. Finally, lactic acid production capacity and the expression of the gene associated with biofilm formation were evaluated. The null hypothesis is that Emo-CS-NPs-mediated aPDT can affect the biofilm and metabolic activities of *S. mutans* by producing intracellular ROS. Also, aPDT influences the lactic acid production, eDNA level, and the *gtfB* gene expression in *S. mutans* biofilms.

## Materials and methods

### Experimental Materials

*S. mutans* strain was purchased from the Iranian Biological Resource Center, Tehran, Iran. Brain heart infusion (BHI) broth, BHI agar, and sodium acetate were supplied by Merck, Germany. Emo and CS were obtained from Sigma-Aldrich, Germany.

### Ethics statement

All experiments have been approved by the Ethics Committee of Tehran University of Medical Sciences (Application No. IR.TUMS.MEDICINE.REC. 1400.090).

### Bacterial growth conditions

The *S. mutans* strain used in this study corresponded to the American Type Culture Collection (ATCC), which is an international reference of ATCC 35,668. The *S. mutans* suspension was prepared by *S. mutans* colonies which were inoculated BHI broth at 37 °C under capnophilic conditions (5% CO_2_). Turbidity density was standardized with half McFarland standard, equivalent to a suspension of 1.5 × 10^8^ bacteria per milliliter.

### Determination of the synergistic effect of Emo and CS by checkerboard array assay

The synergistic effect of Emo and CS combinations was evaluated with the checkerboard assay and determination of the fractional inhibitory concentration (FIC), as previously reported [25, 26]. For the checkerboard assay, the minimum inhibitory concentrations (MIC) of Emo (at a final concentration of 12.5 µg/mL) [16] and CS (at a final concentration of 50 µg/mL) [21] were determined alone and in combinations against *S. mutans* in one 96-well microtiter plate. The FIC index was determined as follows:

$${\mathrm{FIC}}_{\mathrm{index}}=\frac{{\mathrm{MIC}}_{\mathrm{A}}^{\mathrm{ Combination}}}{{\mathrm{MIC}}_{\mathrm{A}}^{\mathrm{ Alone}}}+\frac{{\mathrm{MIC}}_{\mathrm{B}}^{\mathrm{ Cobmbination}}}{{\mathrm{MIC}}_{\mathrm{B}}^{\mathrm{ Alone}}}$$

Generally, the FIC index was interpreted as follows: ≤ 0.5: synergistic effect, > 0.5 to 4: additive or indifferent effect, > 4: antagonism effect.

### Preparation of Emo-CS-NPs

Emo-CS-NPs were prepared based on Jiang et al. study [27] with slight modifications. Briefly, 1 mL of CS at the concentration of MIC combination was stirred for 10 min at room temperature. The Emo solution at the concentration of MIC combination was then slowly added in a drop-wise manner to the CS in an ice bath. The mixture was sonicated by an ultrasonic probe at a power of 400 W for 20 cycles (work 2 s, stop 2 s). The obtained solution was stirred for 30 min and pure Emo-CS-NPs were obtained by centrifugation at 10,000 rpm for 10 min. The final solution was passed through 0.22 μm filters and kept at 4 °C before use.

### Characterization of Emo-CS-NPs

The particle size, zeta potential, and electrophoretic mobility distribution of prepared nanoparticles were characterized by a MALVERN Zetasizer Ver. 6.01 (Malvern Instruments, UK). The morphology of Emo-CS-NPs was observed and photographed by transmission electron microscopy (TEM; ZEISS EM10C, Germany) with an accelerating voltage of 100 kV. The size and aggregation phenomena of Emo-CS-NPs were measured by field emission scanning electron microscopy (FESEM; ZEISS, Germany) under the voltage of 15 kV. Energy dispersive spectroscopy (EDS) mapping was used to show the presence of chemical elements in the Emo-CS-NPs structure. Fourier-transform infrared (FTIR) analysis was performed using a spectrum of two spectrophotometers (45° ZnSe crystal, PerkinElmer Inc., US), within the range of 500–4000 cm^−1^. Proton nuclear magnetic resonance spectra (^1^H NMR) spectra of Emo-CS-NPs were recorded on a Bruker Avance 400 MHz NMR spectrometer. Also, the entrapment efficiency (EE%) and drug loading (DL%) of Emo-CS-NPs were determined by an ultraviolet–visible (UV–vis) spectroscopy (Eppendorf BioSpectrometer®, Germany) at 438 nm following equations:

$$\mathrm E\mathrm E\%=\frac{\mathrm{total}\;\mathrm{amount}\;\mathrm{of}\;\mathrm{Emo}-\mathrm{free}\;\mathrm{Emo}}{\mathrm{total}\;\mathrm{amount}\;\mathrm{of}\;\mathrm{Emo}}\times100$$

and

$$\mathrm D\mathrm L\%=\frac{\mathrm{total}\;\mathrm{amount}\;\mathrm{of}\;\mathrm{Emo}-\mathrm{free}\;\mathrm{Emo}}{\mathrm{Emo}-\mathrm{CS}-\mathrm{NPs}\;\mathrm{weight}}\times100$$

### In vitro drug release

The in vitro release study of Emo from Emo-CS-NPs in different pH was investigated using measurements remained Emo in the supernatant after centrifuging the particles. First, 15 mg of Emo-CS-NPs was dissolved at 10 mL of phosphate-buffered saline (PBS) under different pH conditions (pH 5.5, 7.2, and 8.5) at 37 ± 0.5 °C. At predetermined time intervals, 4 mL of the sample was centrifuged at 3000 rpm for 10 min. The supernatant was then removed and replaced with an equal volume of fresh PBS solution. Subsequently, a UV–Vis spectrophotometer at 430 nm was used to determine the amount of Emo released in the supernatant. The concentration of the released Emo was determined by the free Emo standard curve. The percentage of released Emo under pH 5.5, 7.2, and 8.5 at a specific time was determined based on the following equation:

$$\mathrm{Relesae of Emo }(\mathrm{\%}) =\frac{\mathrm{Emo released}}{\mathrm{Emo loaded in nanoparticles}} \times 100$$

### Evaluation effect of blue laser light on *S. mutans* in the planktonic phase

A blue laser (Laser Diode, Asha, Iran) in a continuous beam was used as a light source with an output intensity of 150 mW/cm^2^, 4.2 V, and 0.34 A at the wavelength of 405 ± 10 nm. To determine the sub-significant reduction dose of blue laser irradiation time, 100 μL of bacterial cells at a final concentration of 1.5 × 10^5^ CFU/mL was exposed to the blue lase at five different times as following 1, 2, 3, 4, and 5 min. Then, 10 μL of each well-contained dilution series were spread onto BHI agar (Merck, Germany). The number of CFU/mL was determined according to the previous study [28].

### Uptake of Emo-CS-NPs in *S. mutans*

The bacterial uptake of Emo, CS, and Emo-CS-NPs was evaluated to determine the incubation time at which the uptake of antimicrobial agents is highest to induce a greater aPDT result [29]. Briefly, 100 μL of *S. mutans* at a final concentration of 1.5 × 10^8^ cells per well was added to the wells of the 96-well microtiter plate and incubated at 37 °C with 5% CO_2_ for 24 h. *S. mutans* were then treated with 100 μg/mL of Emo, CS, and Emo-CS-NPs. At predetermined time intervals, the bacterial cells were washed and uptake of Emo, CS, and Emo-CS-NPs in *S. mutans* was measured using a spectrophotometer at an excitation wavelength of 488 nm and an emission wavelength of 535 nm.

### Preparation of enamel slab

In this study, human premolars without visible cracks and white spot lesions in the buccal and lingual enamel surfaces extracted for orthodontic purposes were selected. The teeth were disinfected and stored in 0.1% thymol solution at 4 °C before use. Enamel slabs measuring 3 × 3 × 1 mm were prepared by cutting thin sections of enamel using a water-cooled carborundum disc. The outer surface of the enamel slabs was polished and about 100 μm of the outermost enamel layer was then removed during the polishing process [30].

### Biofilm formation on enamel slabs

As Pourhajibagher and Bahador reported [31], *S. mutans* biofilms were formed on enamel slabs. Briefly, the slabs were placed into the 48-well microtiter plate containing *S. mutans* suspension (at the concentration of 1.5 × 10^8^ CFU/mL) supplemented with filtered human saliva and incubated at 37 °C for one week. The culture medium was changed every 24 h and the bacterial biofilms were treated with sucrose (10%). The biofilms were treated as mentioned in the experimental study section.

### Experimental design and treatment procedure

The enamel slabs covered with microbial biofilms were randomly divided into the experimental groups (*n* = 5) as follow:

A **Emo:** 300 μL of Emo at sub-MIC dose was added to the enamel slabs and the samples were incubated in the dark at room temperature for 5 min to correlate maximum uptake of Emo by *S. mutans* according to the uptake measurements section.
B **CS:** 300 μL of CS at sub-MIC dose was added to the enamel slabs and the samples were incubated in the dark at room temperature for 5 min to correlate maximum uptake of CS by *S. mutans*.
C **Emo-CS-NPs:** 300 μL of Emo-CS-NPs at sub-MIC dose was added to the enamel slabs. After that the samples were incubated in the dark at room temperature for 5 min to correlate maximum uptake of Emo-CS-NPs by *S. mutans*.
D **Blue laser:** The enamel slabs were exposed with a sub-significant reduction dose of blue laser irradiation time at the wavelength of 405 ± 10 nm and output intensity of 150 mW/cm^2^. The optical fiber reached up to 2 cm shorter than the working length.
E **aPDT:** *S. mutans *biofilms on the enamel slabs were separately treated by Emo, CS, and Emo-CS-NPs similar to groups A-C, respectively. Then the samples were exposed with a sub-significant reduction dose of blue laser similar to group D.
F **Positive control:** 300 μL of 0.2% chlorhexidine (CHX) was added to the enamel slabs and the samples were incubated in the dark at room temperature for 5 min.
G **Negative control:** 300 μL of normal saline was added to the enamel slabs and the samples were incubated in the dark at room temperature for 5 min.

### Assessment of biofilm disruption in enamel slab model using a colorimetric assay

A colorimetric assay was performed according to He et al. modified method [32]. Briefly, the enamel slabs treated with the groups of experimental design section were washed using phosphate-buffered saline (PBS) and stained with 200 μL of 0.1% crystal violet. After 10 min, the enamel slabs were washed by PBS and were placed in the wells of microtiter plates. The 96% ethanol solution was employed to solubilize the crystal violet-stained slabs. The absorbance of the solution containing crystal violet was then measured at 570 nm by a microplate reader. The percentage of biofilm degradation was determined as follows:

$$\mathrm{Biofilm degradation \% }=\frac{\mathrm{OD of untreated slab}-\mathrm{ OD of treated slab}}{\mathrm{OD of untreated slab}} \times 100$$

### Assessment of biofilm dispersal in enamel slab model

The biofilm dispersal assays were done as described previously [33]. Briefly, the 24-h-old mature biofilms were prepared as described for our biofilm formation on enamel slabs. Then all non-attached bacterial cells on enamel slabs were removed by rinsing the enamel slabs in a container by immersing and agitating gently four times in sterile PBS. Enamel slabs covered with microbial biofilms were transferred to wells of the new sterile 48-well microtiter plate. After adding 300 μL of fresh medium to each well, each treatment was done according to the study design. The culture medium was then carefully obtained from each well without disturbing the attached biofilm to the enamel slabs and transferred to the new 96-well microtiter plate. Eventually, the optical density of the medium was read at 600 nm.

### Determination of intracellular ROS production

According to the previous study [34], 2′-7′-dichlorodihydrofluorescein diacetate (DCFH-DA) (Sigma-Aldrich, United Kingdom) was used to determine the intracellular ROS generation. Briefly, 100 μL of *S. mutans* at a final concentration of 1.5 × 10^8^ cells per well was mixed with 100 μL of 5 μM DCFH-DA solubilized in 96% ethanol into a 96-well microtiter plate. After 60 min of incubation at 37 °C, the microbial cells were centrifuged at 5000 rpm for 15 min and the pellets were washed with PBS. The cells were then treated based on the experimental design section and the samples were subjected to fluorescence spectrophotometric analysis at an excitation wavelength of 488 nm and an emission wavelength of 535 nm.

### Evaluation of eDNA levels

eDNA of *S. mutans* was assessed as previously described by Kim et al. [35] with some modifications. Briefly, *S. mutans* was cultured in BHI agar supplemented with 1% sucrose for 48 h. After treatment of the microbial biofilms based on the experimental design section, the bacterial cells on the wells were washed and collected in PBS by scraping them from the wells using a spatula. The bacteria cells were centrifuged at 10,000 × g at 4 °C for 10 min, and the supernatant was filtered using 0.22-μm syringe filters. The cell-free supernatants were then mixed with three volumes of absolute ethanol and a 1/10 volume of 3 M sodium acetate (pH 5.5). After incubation of samples at -80 °C overnight, eDNA was collected by centrifugation at 13,000 × g for 20 min, washed twice with ice-cold 70% ethanol, air dried, and then dissolved in sterile TE buffer (TrisHCl/1 mM EDTA, pH 8.0). eDNA was measured by spectrophotometry at an emission wavelength of 535 nm and an excitation wavelength of 485 nm.

### Determination of lactic acid production

The enamel slabs treated with the groups of experimental design section were rinsed in cysteine peptone water and transferred to a 48-well microtiter plate containing 0.5 mL of buffered peptone water supplemented with 0.2% sucrose. The enamel slabs were then incubated at 5% CO2 and 37 °C to allow the acid generation. After 3 h, the lactic acid production in the buffered peptone water and the absorbance of collected buffered peptone water solutions were measured by an enzymatic (lactate dehydrogenase) method and a microplate reader at 340 nm, respectively [36].

### Evaluation of *S. mutans* metabolic activity using XTT reduction assay

The metabolic activity of *S. mutans* on the treated enamel slabs was evaluated using the XTT (2,3-bis [2-methyloxy-4-nitro-5-sulfophenyl]-2H-tetrazolium-5-carboxanilide) reduction assay kit (Roche Applied Science, Indianapolis, IN, US) as Coraça-Hubér et al. described [37]. Briefly, the treated *S. mutans* biofilms were sonicated for 10 s at the frequency and output power of 50 kHz and 2.5 W, respectively. After centrifuging the microbial suspensions at 2000 rpm for 10 min, the microbial cell sediments were collected, dissolved in 200 μL of XTT-menadione-PBS solution, and incubated for 3 h at 37 °C. Half of the solution was transferred to a new 96-well microtiter plate and the absorbance was then measured at 492 nm using a microplate reader. The rest of the suspension was used to assess changes in gene expression.

### Effect of aPDT using Emo-CS-NPs on *gtfB* mRNA expression

The super RNA extraction Kit (AnaCell, Iran) was used to obtain total RNA from treated *S. mutans*. Total RNA (200 ng) was reverse transcribed in a 20 μL cDNA reaction volume RevertAid First Strand cDNA Synthesis Kit (Thermo Scientific GmbH, Germany) based on the manufacturer’s instructions. Real-time RT-PCR analysis was then performed using SYBR® Premix Ex Raq™ II (TliRNaseH Plus; Takara, Korea) on Line-GeneK Real-Time PCR Detection System and Software (Bioer Technology, Hangzhou, China). The sequences of forward and reverse primers of *gtfB* gene and the thermal cycling conditions were similar to our previous study [38]. Fold differences in RNA expression were determined by the 2^−ΔΔCt^ method and the changes greater than or equal to two-fold were considered significant [39].

### Statistical analyses

All data are expressed in mean ± standard deviation (SD) after triplicate sample analysis. The results were statistically evaluated by one-way analysis of variance (ANOVA) and a value of *P* < 0.05 was considered to be statistically significant.

## Results

### The synergistic effect of nEmo and CS

**Fig. 1:**
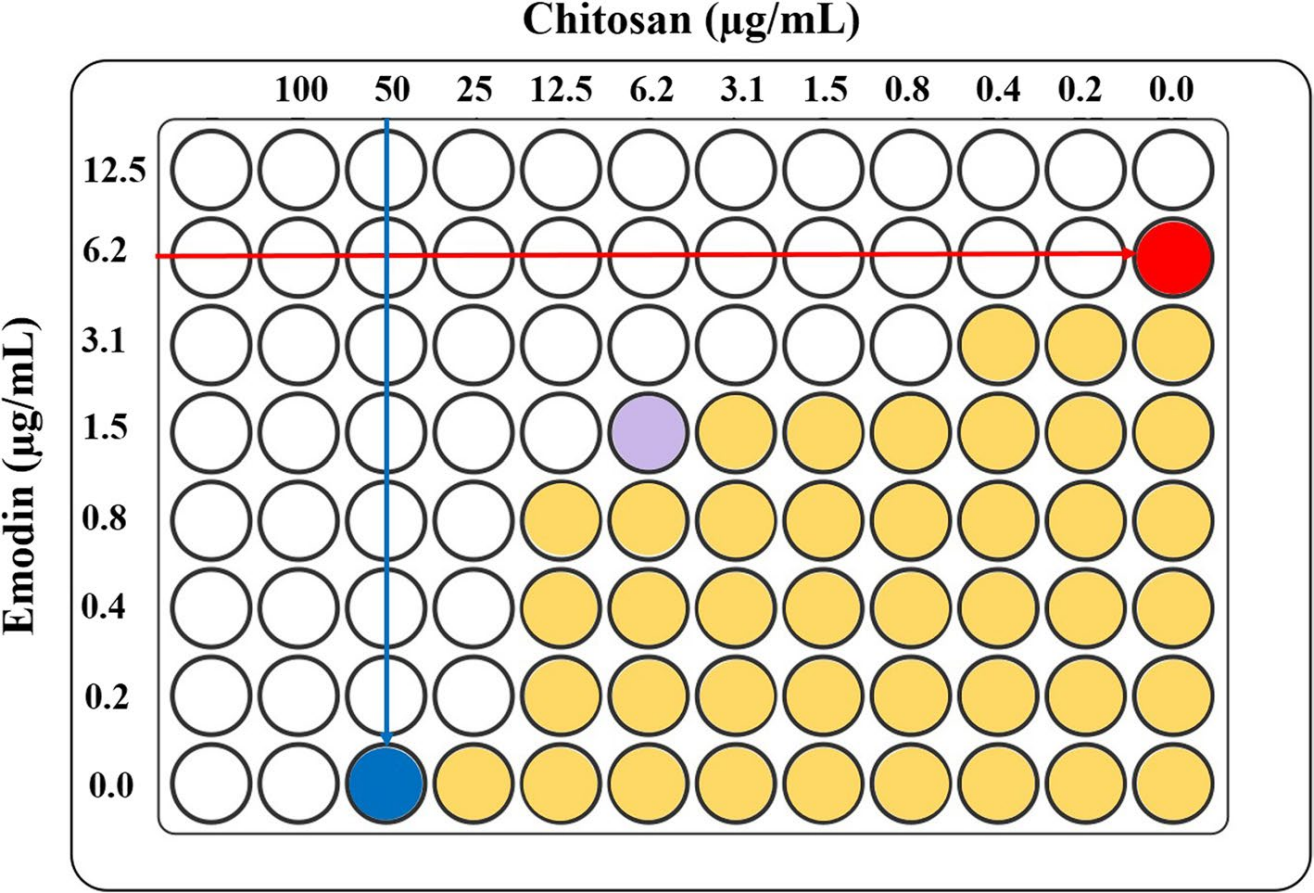
Checkerboard titration to evaluate antimicrobial synergism (Emodin + Chitosan tested against *Streptococcus mutans*).  Minimum inhibitory concentration (MIC) of emodin,  MIC of chitosan,  MIC of Emodin + Chitosan

As shown in Fig. 1, the MICs of Emo and CS were 6.2 and 50 µg/mL, respectively. According to the checkerboard array assay, the FIC index value was 0.36 in Emo-CS-NPs, and synergism (FIC Index ≤ 0.5) was confirmed.

### Confirmation of Emo-CS-NPs synthesis

**Fig. 2:**
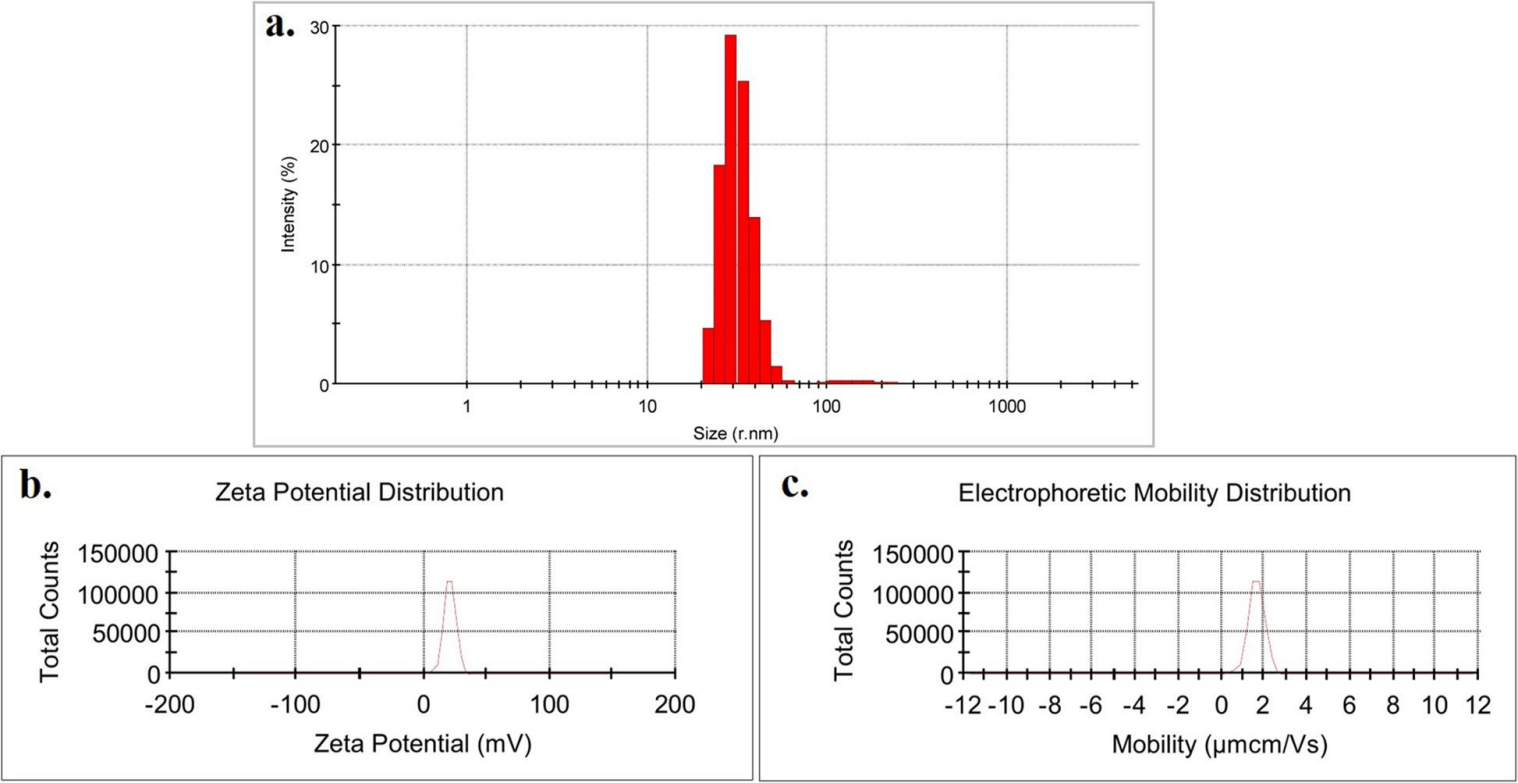
Characterization of emodin-chitosan nanoparticles: a) Size characterization by DLS; b) Zeta potential; and c) Electrophoretic mobility distribution

The results in Fig. 2 show that the average diameter of Emo-CS-NPs was 35.34 ± 5.6 nm (a). Their surface charge (b) and electrophoretic mobility distribution (c) were 20.8 ± 4.86 mV and 1.632 ± 0.38 µmcm/Vs, respectively.

**Fig. 3:**
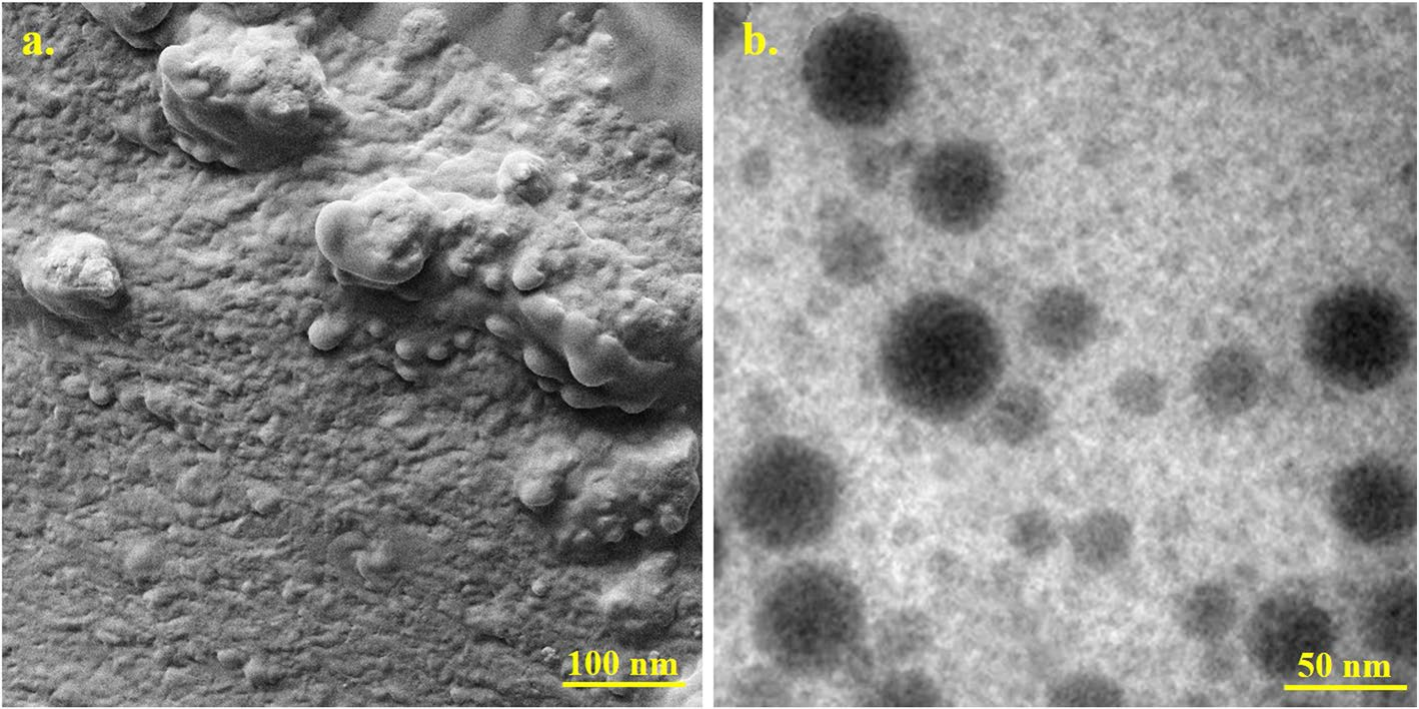
**a** Field emission scanning electron microscopy (FESEM) image of emodin-chitosan-nanoparticles, Scale bar = 100 nm; **b** Transmission electron microscopy (TEM) micrograph of emodin-chitosan-nanoparticles. Scale bar = 50 nm

As demonstrated in Fig. 3a, the typical spherical shape of Emo-CS-NPs was visible and the distribution of particles was visually acceptable. Also, TEM confirmed the round and uniform shape of Emo-CS-NPs (Fig. 3b).

**Fig. 4:**
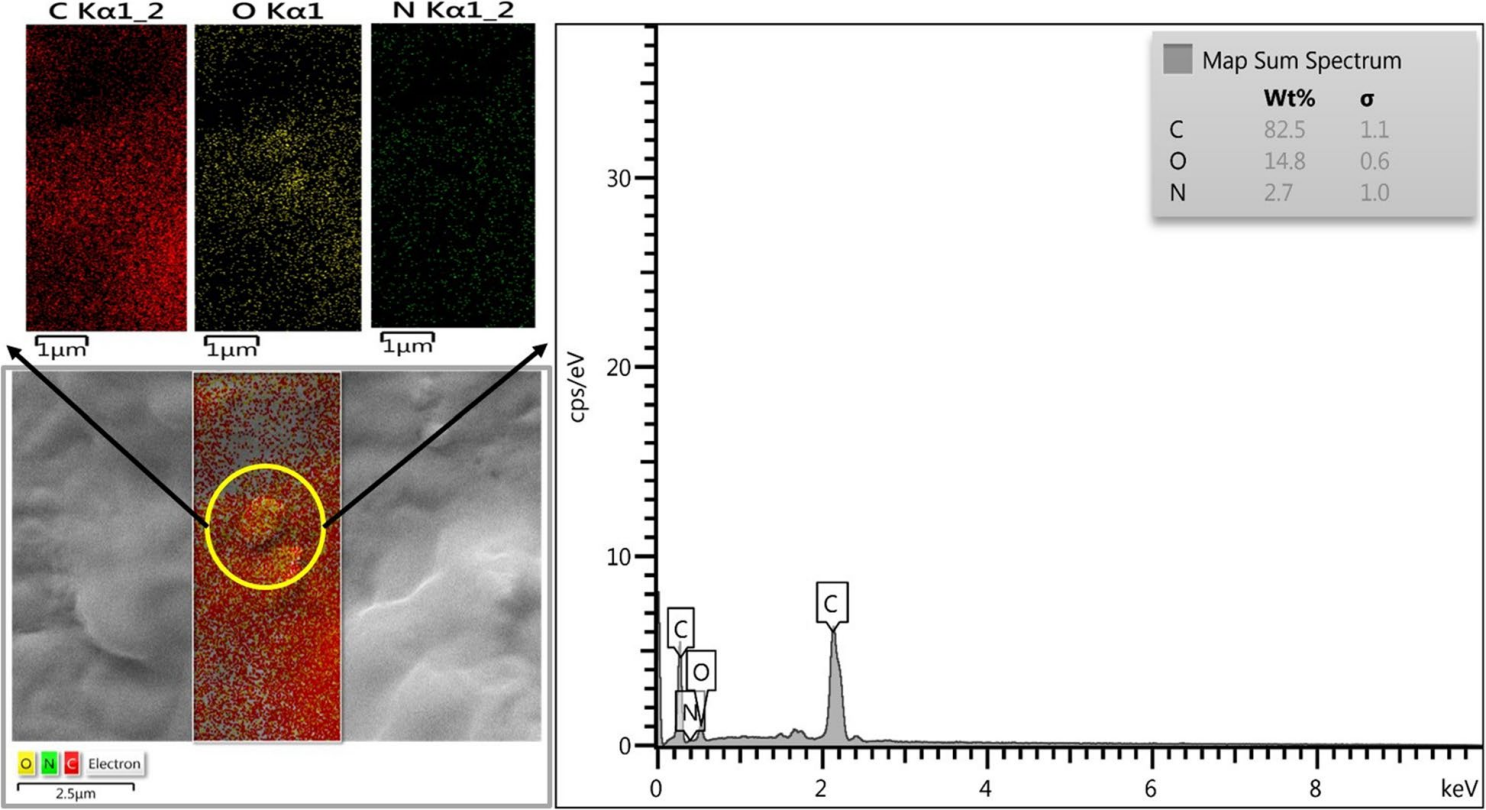
Energy dispersive spectroscopy (EDS) spectrum of emodin-chitosan-nanoparticles

According to the results in Fig. 4, the elemental mapping of Emo-CS-NPs shows a homogeneous distribution of the mentioned elements with the percentage of each element over the entire nanophotosensitizer. In addition, the EE and DL percentage of the Emo in Emo-CS-NPs were 63.2 ± 4.05% and 7.51 ± 0.21%, respectively.

**Fig. 5:**
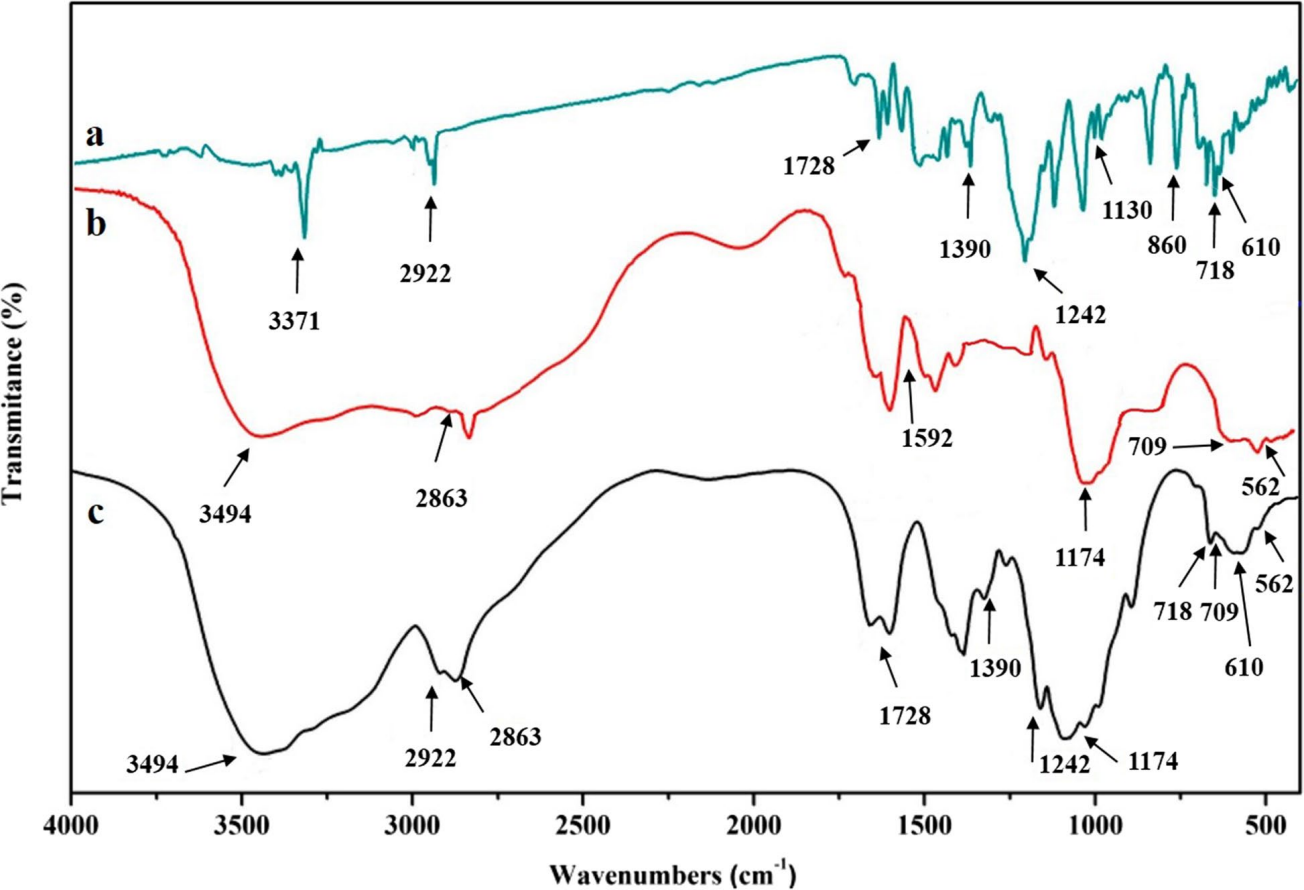
FTIR spectra of (**a**) chitosan, (**b**) emodin, and (**C**) emodin-chitosan nanoparticles

The FTIR spectra of Emo, CS, and Emo-CS-NPs were shown in Fig. 5. In the Emo spectrum, the peaks at 610 cm^−1^, 718 cm^−1^, and 860 cm^−1^could be belonged to C–H and C–C stretching. The peaks at 1130 cm^−1^, 1242 cm^−1^, and 1390 cm^−1^ can be assigned to C–O bond stretching vibration. The C–H stretching shows a peak at 2922 cm^−1^ which belongs to the alkyl group. The broad band at 3371 cm^−1^ may be attributed to O–H stretching because of the presence of the hydroxyl group of Emo. The FTIR spectra of CS showed the peak at 562 cm^−1^ correspond to out-of-plane bending C–O, whereas those at 709 cm^−1^ and 1174 cm^−1^ were associated with out-of-plane bending NH and C–O–C stretching, respectively. The vibrational mode of C = O stretching was observed at 1592 cm^−1^. The peaks at 2863 cm^−1^ and 3494 cm^−1^ indicated the CH_2_ stretching and − OH stretching, respectively. As expected, no new peak appeared in Emo-CS-NPs confirming that there was no interfacial interaction and chemical bonding between Emo and CS.

**Fig. 6:**
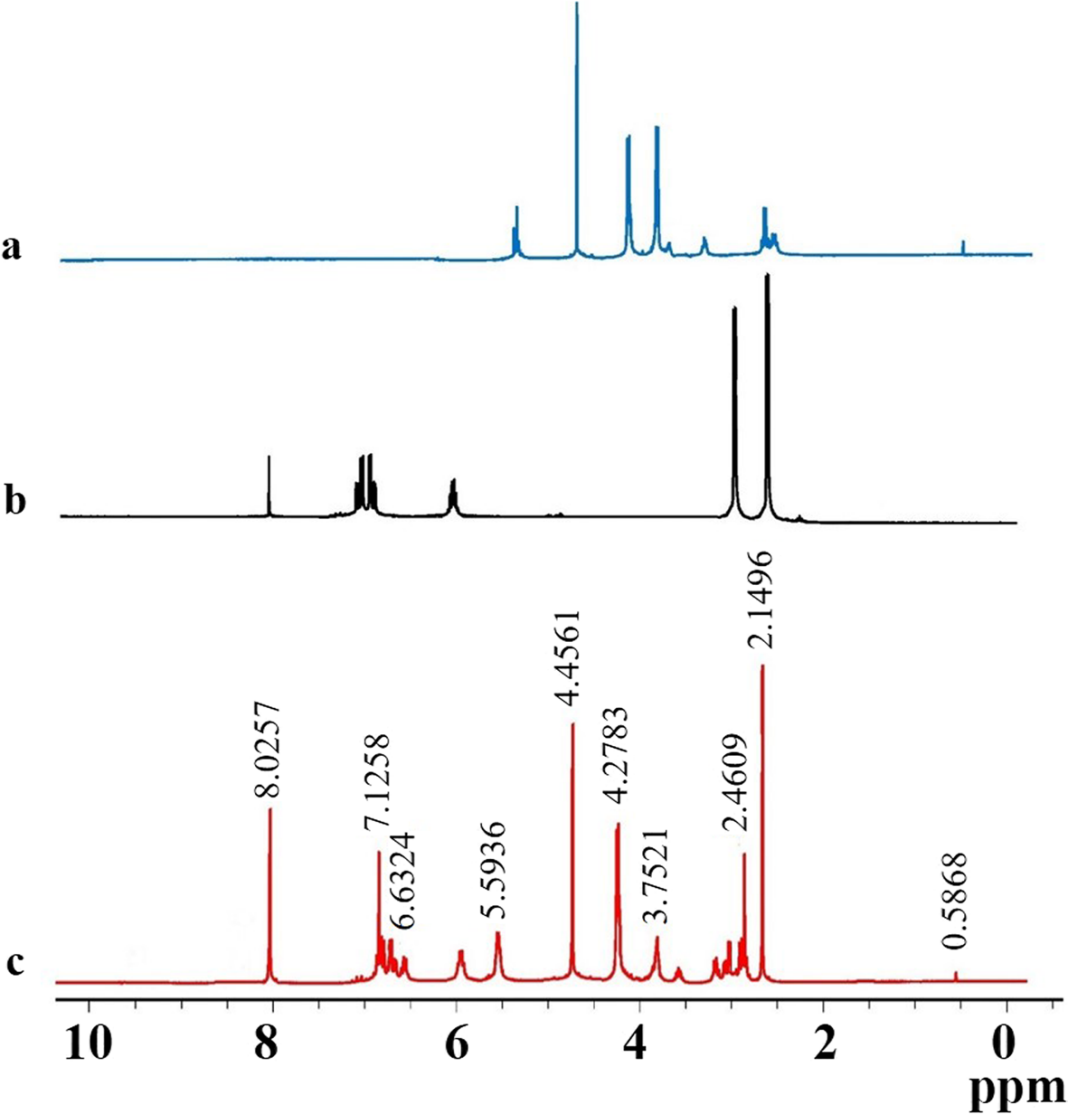
^1^H NMR spectra of (**a**) chitosan, (**b**) emodin, and (**C**) emodin-chitosan nanoparticles

The ^1^H NMR spectra of EM, CS, and Emo-CS-NPs are shown in Fig. 6. The NMR analysis reveals that the non-overlapping signal could be observed in the 1H NMR spectrum of the sample.

### In vitro drug release

**Fig. 7:**
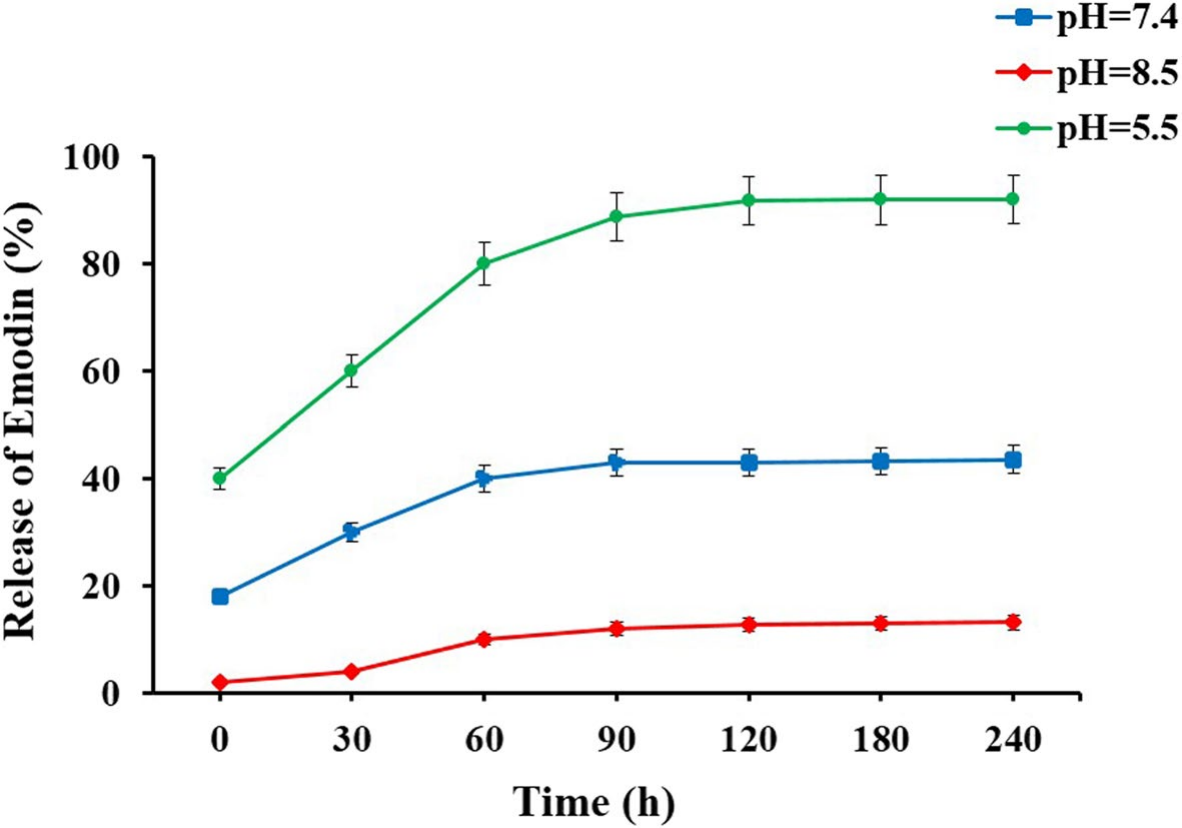
pH-responsive release profiles of emodin from emodin-chitosan nanoparticles under different pH values

The release characteristics of Emo from Emo-CS-NPs were shown in Fig. 7. According to the findings, a total of about 13% Emo was released gradually out from Emo-CS-NPs during 240 h of incubation under an alkaline condition at 37 °C. Also, a slow-release behavior with less than ∼45% released Emo detected in aqueous solution at pH 7.2 (a neutral condition). However, up to over ∼ 90% of Emo had been released within a period of 240 h in weak-acid environment (pH 5.5). Consequently, the pH-responsive Emo-CS-NPs-delivery system could facilitate the accumulation of Emo-CS-NPs in bacterial cells and hence greatly improve the anti-biofilm activity of photodynamic therapy against *S. mutans* biofilms.

### Efficacy of blue laser light on *S. mutans*

**Fig. 8:**
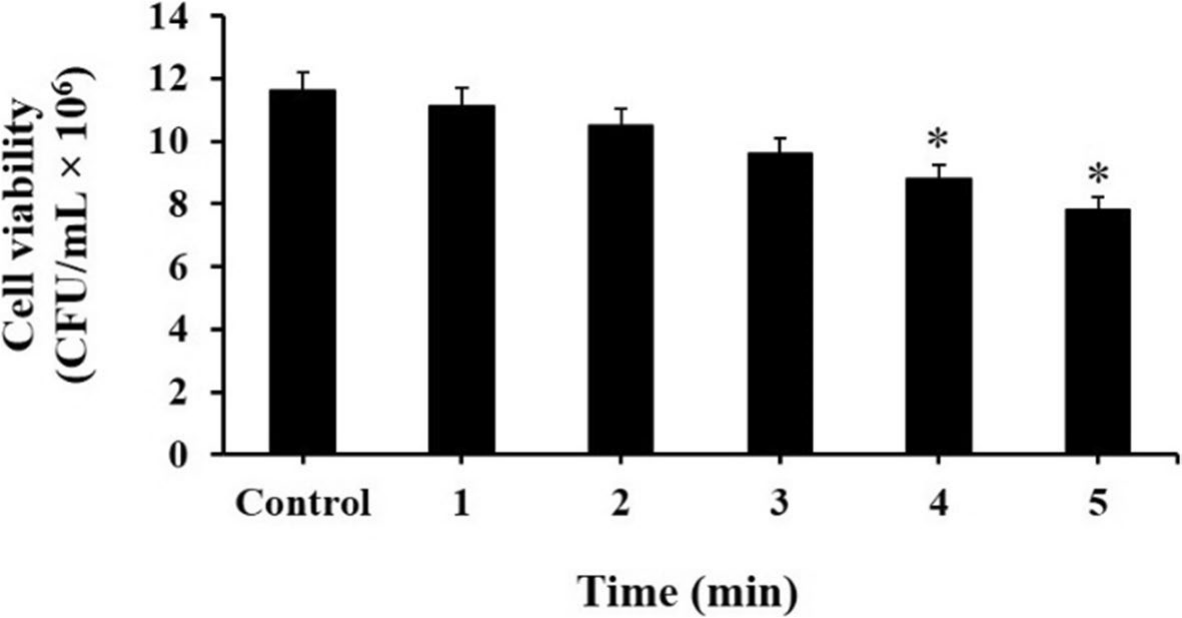
Effect of different times of blue laser irradiation on cell viability of *Streptococcus mutans.* *Significantly different from the control group (no treatment), *P* < 0.05

The cell viability of *S. mutans* was determined after exposure to blue laser at different irradiation times. As seen in Fig. 8, irradiation times of 1, 2, and 3 min with energy doses of 104.5, 209.1, and 313.7 J/cm^2^, respectively had no effect on viable cells (4.0%, 9.5%, and 17.2% reduction, respectively); it was nearly the same as the untreated control group (*P* > 0.05). In contrast, blue laser light with energy doses of 418 and 522.8 J/cm^2^ (irradiation times of 4 and 5 min, respectively) resulted in a statistically significant amount of cell reduction (24.1% and 32.6%, respectively; *P* < 0.05). So, 3 min of light irradiation was considered as the maximum sub-significant reduction dose of blue laser irradiation time.

### Cellular uptake measurements

**Fig. 9:**
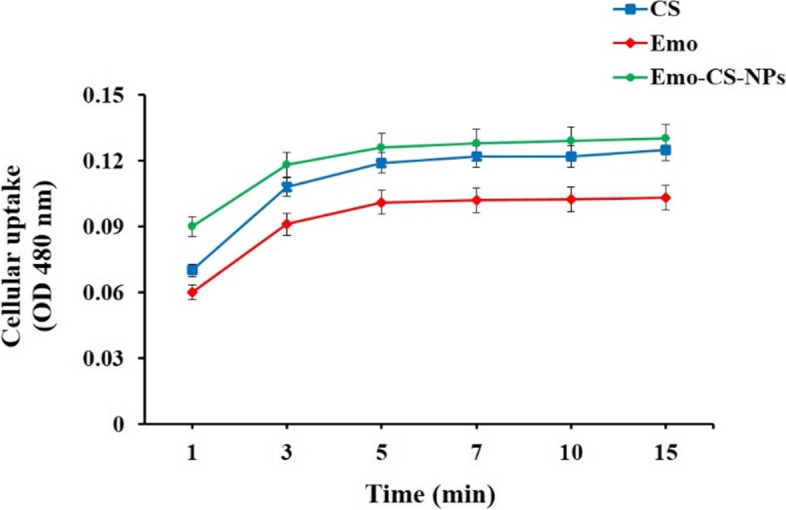
Cellular uptake of emodin, chitosan, and emodin-chitosan-nanoparticles by *Streptococcus mutans*

The uptake times of Emo, CS, and Emo-CS-NPs by *S. mutans* cells were determined following 30 min incubation. According to the results in Fig. 9, Emo-CS-NPs were rapidly taken up by *S. mutans* cell being observed in the first 3 min and gradually increased through time and reached plateau phase at about 5 min. The uptakes of Emo and CS by *S. mutans* were similar to Emo-CS-NPs in a time-dependent manner and no significant difference was observed between them (*P* > 0.05).

### Biofilm disruption effects of treatment groups against *S. mutans*

**Fig. 10:**
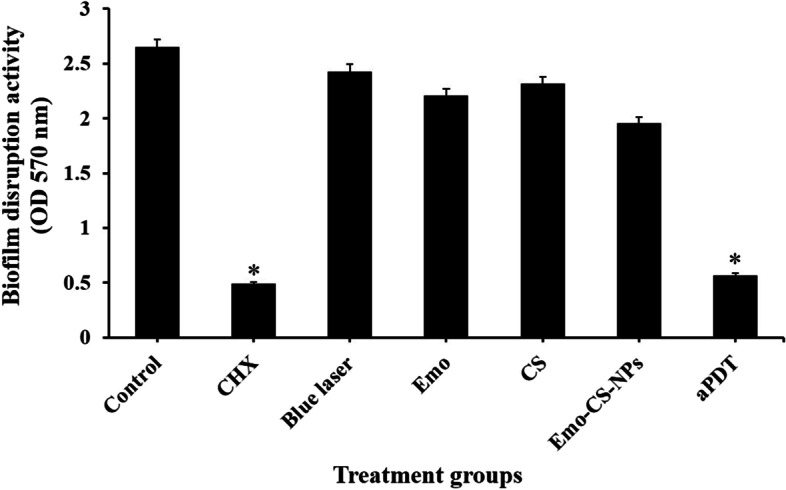
Biofilm disruption effects of different treatment groups against *S. mutans.* *Significantly different from the control group (no treatment), *P* < 0.05

The crystal violet results in Fig. 10 revealed that the aPDT based on sub-MIC of Emo-CS-NPs (0.58 µg/mL Emo/ 3.1 µg/mL CS) plus maximum sub-significant reduction dose of blue laser light (3 min, 313.7 J/cm^2^) had significantly anti-biofilm effects against *S. mutans* in comparison with the control group (*P* < 0.05). The biofilms of *S. mutans* showed to be more susceptible to Emo than CS, however, this difference is not significant (*P* > 0.05). As the results showed, there was no significant reduction effect of blue laser light against *S. mutans* biofilms (8.3%) versus the control group (*P* > 0.05). Although the biofilm reduction activity of CHX against *S. mutans* biofilms (81.5%) was significantly higher than the other treatment groups. However, the rate of biofilm reduction in aPDT group was roughly close to 0.2% CHX (*P* > 0.05).

### Biofilm dispersal effects of treatment groups against *S. mutans*

**Fig. 11:**
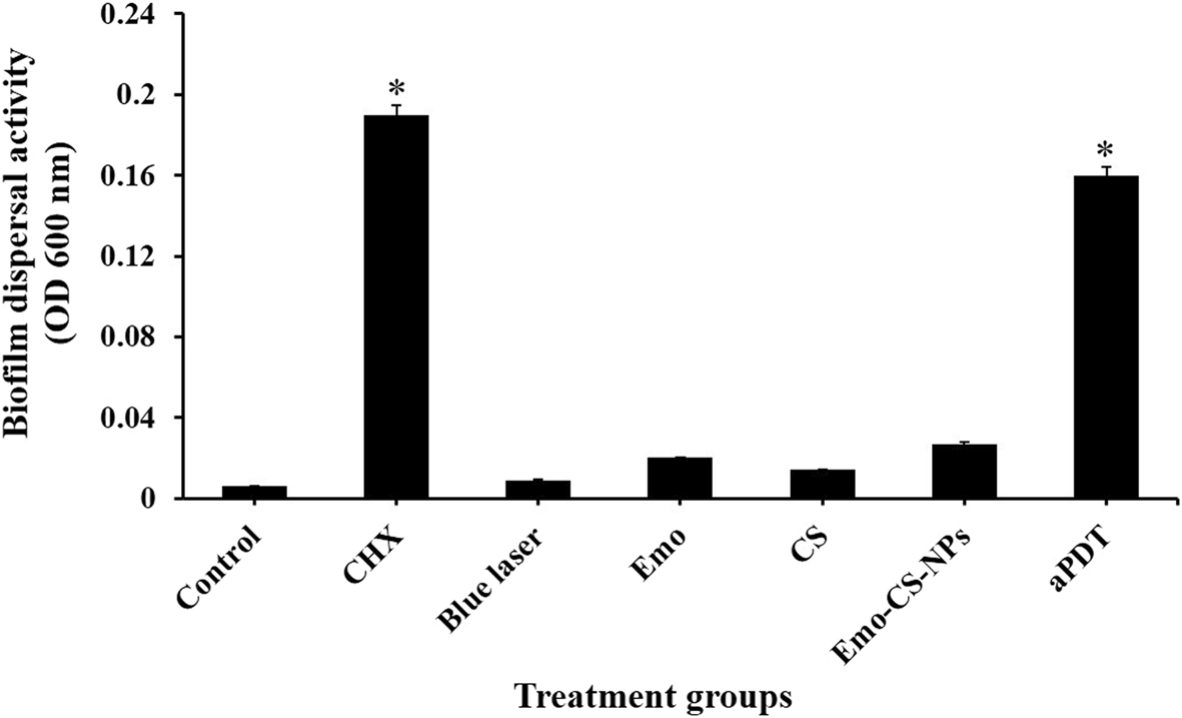
Biofilm dispersal effects of different treatment groups against *S. mutans.* *Significantly different from the control group (no treatment), *P* < 0.05

Emo-CS-NPs-mediated aPDT and 0.2% CHX revealed high dispersal effects (26.6- and 31.6-fold, respectively) on the bacterial biofilm in comparison with the control group (Fig. 11). There was low dispersal for Emo and CS groups (3.3- and 2.3-fold, respectively). We also tested the activity of combination Emo plus CS (Emo-CS-NPs), but no significant increase in dispersal was found compared to Emo or CS alone. No dispersal was observed using the blue laser at the intensity of 150 mW/cm^2^.

### Measurement of intracellular ROS generation in *S. mutans*

**Fig. 12:**
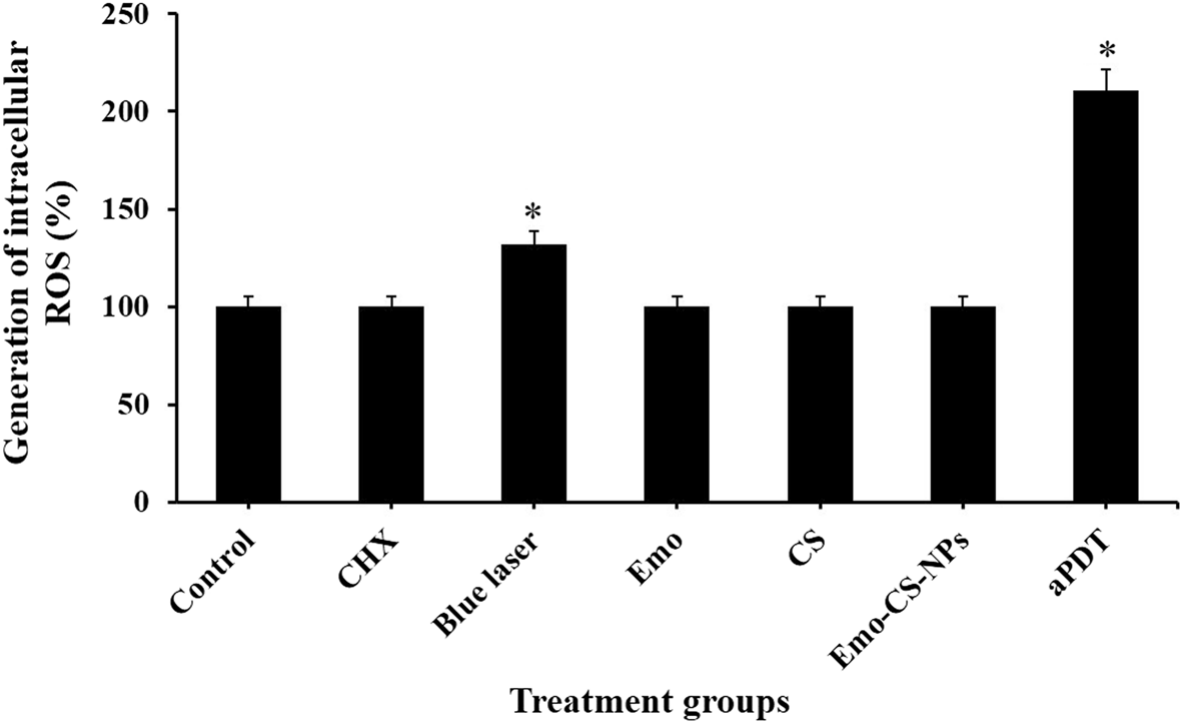
Effects of different treatment groups on the generation of intracellular reactive oxygen species (ROS) in *Streptococcus mutans.* *Significantly different from the control group (no treatment), *P* < 0.05

There was no considerable increase in the DCFH-DA fluorescence in *S. mutans* cells subjected to Emo, CS, and Emo-CS-NPs alone (*P* > 0.05). A rise (1.32-fold) in the fluorescence was observed in *S. mutans* exposed to blue laser irradiation alone compared to the control group (Fig. 12; *P* < 0.05). Most notably, the highest amount of ROS generation was evident when *S. mutans* cells were subjected to Emo-CS-NPs-mediated aPDT (2.11-fold; *P* < 0.05).

### Effects of treatment groups on *S. mutans* eDNA and lactic acid production

**Fig. 13:**
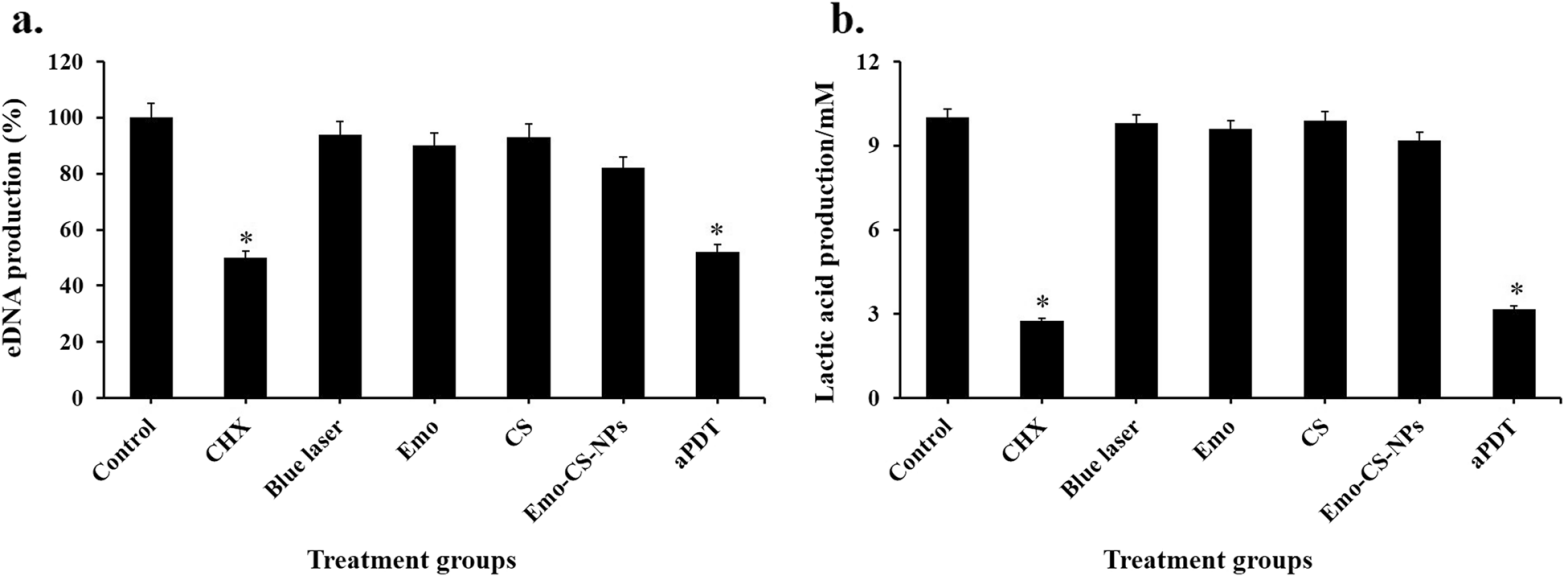
Effects of different treatment groups on **a** extracellular DNA (eDNA) levels and **b** lactic acid production capacity of *Streptococcus mutans.* *Significantly different from the control group (no treatment), *P* < 0.05

No significant reduction was observed in eDNA production under the conditions studied, except aPDT and CHX (Fig. 13a). Emo-CS-NPs-mediated aPDT led to a significant reduction of eDNA levels (*P* < 0.05). The eDNA levels were approximately 1.09- and 1.13-fold higher in Emo and CS than Emo-CS-NPs and about 1.04- and 1.01-fold lower than blue laser, but the differences were not statistically significant (*P* > 0.05). Also, results showed no significant differences between aPDT and CHX in reducing eDNA production (*P* > 0.05).

The lactic acid production results displayed that *S. mutans* treated with aPDT and CHX groups produced less lactic acid than other groups (Fig. 13b, *P*< 0.05). Emo-CS-NPs-mediated aPDT and CHX groups reduced lactic acid production to 72.4% and 68.2%, respectively compared to the control group (*P* < 0.05). Based on the results, no significant reduction in lactic acid production was observed after treatment of *S. mutans* with Emo, CS, and blue laser in comparison with the control group (*P* < 0.05).

### Effects of treatment groups on the metabolic activity of *S. mutans*

**Fig. 14:**
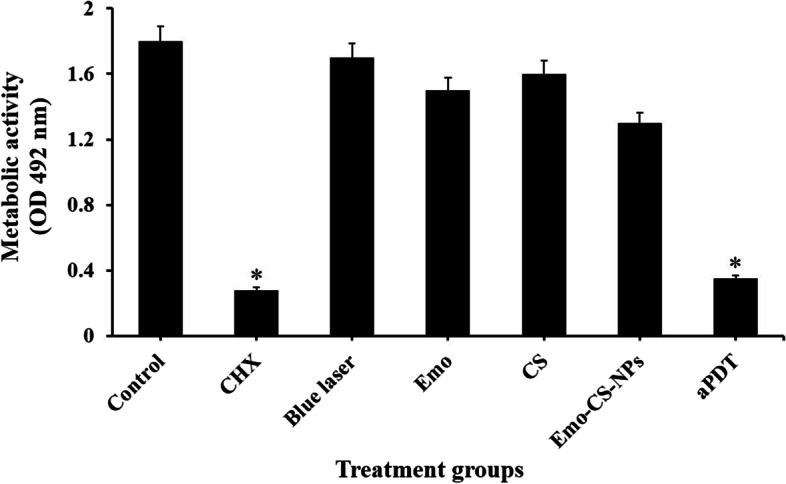
Effects of different treatment groups on the metabolic activity of *Streptococcus mutans.* *Significantly different from the control group (no treatment), *P* < 0.05

The XTT results are plotted in Fig. 14. The *S. mutans* metabolic activity revealed by XTT assay showed a similar trend with biofilm potency. The control group had the highest absorbance. Emo-CS-NPs had an absorbance of 1.15- and 1.23-fold less than that of Emo and CS, respectively. The Emo-CS-NPs based aPDT and CHX groups had the lowest absorbance, which was 5.14- and 6.42-fold less than that of the control group. The OD values in the blue laser group reduced not significantly when compared with the control group (*P* > 0.05).

### Determination of the virulence gene expression patterns in treated *S. mutans*

**Fig. 15:**
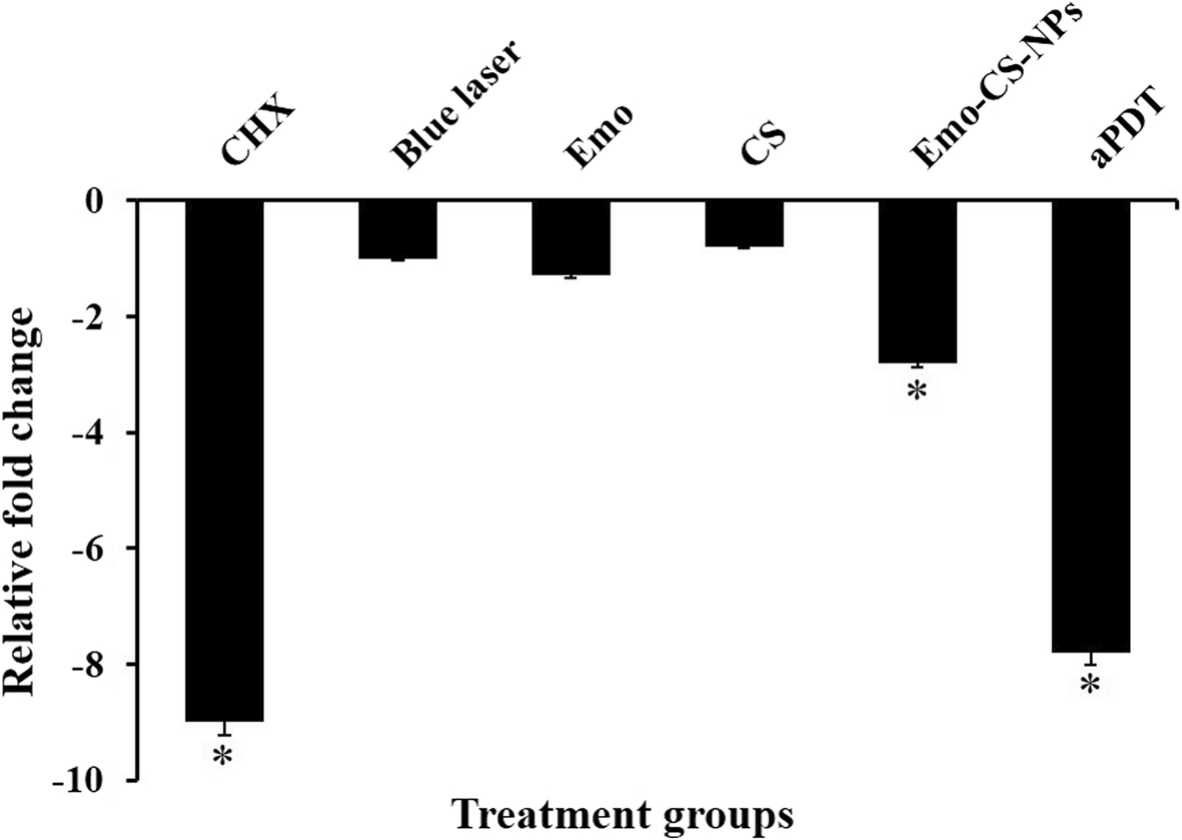
The mRNA expression levels of *gtfB* in *Streptococcus mutans* biofilm following different treatment groups. *Significantly different from the control group (no treatment), *P* < 0.05

Compared with the control group, *gtfB* mRNA expression in *S. mutans* was downregulated after aPDT (Fig. 15; *P* < 0.05). Also, the expression level of *gtfB* in *S. mutans* treated with Emo-CS-NPs was nearly 2.8-fold less than in the control group (*P* < 0.05). There was a statistically decrease in *gtfB* expression to 0.8-, 1.0-, and 1.3-fold following treatment by CS, blue laser, and Emo, respectively, however, these changes were not significant (*P* > 0.05).

## Discussion

In the current study, the *S. mutans* biofilm model on the enamel slabs was degraded following treatment with Emo-CS-NPs based aPDT. The advantages of aPDT approach include non-invasiveness, reproducibility, a wide range of antimicrobial activity, lack of photo-resistant species after multiple treatments, and the ability to localize on the target site [40, 41]. The photosensitizer is one of the most important factors in this method. An ideal photosensitizer should be easily synthesized, have a minimal self-aggregation tendency, be a stable compound, not toxic in the absence of light, and have a good light absorption with a high extinction coefficient [42–44]. Different nanoparticles each have their own characteristics in terms of the type of function. Since the antimicrobial efficacy of aPDT depends on the amount of singlet oxygen generated, two strategies for nanoparticles in aPDT are proposed: (a) degradable nanoparticles that release photosensitizers at target sites and then produce oxygen. b) Non-degradable nanoparticles in which single oxygen is produced and then diffused [45].

The results of Tarsi et al. [46] study have shown that the use of CS with hydroxyapatite as the dental materials increases the biocompatibility and prevents the adherence of *S. mutans* to the tooth surface. Some notable investigated properties of CS in different formulations including mouth rinses, toothpaste, and chewing gums have also been effective as antimicrobial agents in controlling the *S. mutans* [47, 48]. The data in the literature reveal that the effectiveness of CS varies and is dependent on species of target microorganisms in such a way that MIC values found for CS ranged from 20 to 1280 µg/mL [48]. According to the results of this study, the MIC value of CS was 50 µg/mL. On the other hand, Emo inhibits the growth of various strains of microorganisms through the destruction of the cell membrane integrity and increasing membrane permeability [49–54]. Li et al. [49] reported Emo exhibited antibacterial activity against *Haemophilus parasuis* with MIC value of 32 μg/mL. In studies by Lu et al. [50] and Zhou et al. [51], the MIC values of Emo against *Staphylococcus aureus* and *Aeromonas hydrophila* were 20 μg/mL and 50 μg/mL, respectively. In the present study, MIC value of Emo against *S. mutans* was 6.2 μg/mL.

Recently, the advances in nanotechnology, particularly the development of nanophotosensitizer as a targeted drug delivery system, have improved the traditional concepts of aPDT for several main reasons as follows: 1) Enhance photosensitizer concentration and decrease cytotoxic effects on normal tissues and/or cells, 2) Improve the solubility of hydrophobic photosensitizer, and 3) Maintain the constant rate of photosensitizer delivery at target sites [55–57]. In the current study, Emo-CS-NPs were used as a nanophotosensitizer due to their high biocompatibility, stability in diverse biological conditions, and antimicrobial properties.

To the best of our knowledge, the anti-biofilm activity of aPDT using Emo-CS-NPs has not yet been reported in the preformed microbial biofilm model on the enamel surfaces. Results of this study have been shown that aPDT using sub-MIC of Emo-CS-NPs plus maximum sub-significant reduction dose of blue laser light could destroy the microbial biofilm formed on the surface of the enamel slabs.

Blue laser light activated Emo-CS-NPs through the ROS generation could provoke internal osmotic imbalances by promoting changes in the properties of *S. mutans* cell membrane permeability. It also increases the leakage of intracellular electrolytes by the hydrolysis of the peptidoglycans in the *S. mutans* wall. These changes eventually lead to inhibit the biofilm formation of *S. mutans*.

There is evidence that eDNA can enhance the adhesion of *S. mutans* to hydrophobic surfaces and subsequently increase the viscoelasticity of the biofilms [6–8, 58]. aPDT using Emo-CS-NPs led to a significant reduction of eDNA levels. Similar to the Kim et al. [35] results, our foundlings demonstrate that eDNA levels in biofilms are dependent on the CFUs in the biofilms, and the lactic acid production of *S. mutans* biofilms. Also, the results presented here indicate that lactic acid production of biofilm in aPDT group decreased by 72.4% when compared with the control group.

As previously mentioned, the tooth enamel demineralization and dental caries is initiated through the production of organic acids such as lactic acid by microbial plaques. aPDT can reduce and inhibit the tooth enamel demineralization and dental caries via suppuration of the lactic acid production capacity in *S. mutans* biofilms [59, 60]. In this study, lactate dehydrogenase calorimetry was employed to assay the lactate concentration. Compared with the control group, the aPDT treatment group showed reduced acid production capacity of plaque biofilm with a significant difference. Consistent with the results of our study, Liang et al. [59] showed that lactic acid production in all aPDT groups except the photosensitizers (methylene blue and hematoporphyrin monomethyl ether) group was statistically lower than that in the normal saline control group.

*S. mutans* strains produce three genetically separate *gtfs* (*gtfB*, *gtfC*, and *gtfD*) genes that are involved in bacterial cell adhesion and microbial biofilm formation [61]. Our data indicate that Emo-CS-NPs downregulated the gene expression level of *gtfB*, especially during aPDT. No major differences in the reduction of mRNA expression of *S. mutans* were observed between the blue laser alone and the control group. Besides aPDT role in the downregulation of gene expression involved in bacterial virulence, it influences the biofilm metabolic activity of *S. mutans*. aPDT treatment group showed a decrease in the metabolic activity by 80.5% compared to the control group that the difference was statistically significant.

Overall, there appears to be a connection between the employment of Emo-CS-NPs-mediated aPDT with the inhibition of *S. mutans* biofilm activity, the reduction of lactic acid metabolism, the decrease of biofilm metabolic activity, and the downregulation of *gtfB* in the present study.

## Conclusion

It can be concluded that an increase in the anti-biofilm, anti-metabolic, and anti-virulence activities and a decrease in *S. mutans* eDNA and lactic acid production followed by the excessive intracellular ROS generation induced by aPDT in the presence of Emo-Cur-NPs were the main responsible for the significant elimination of *S. mutans* biofilms on the enamel surface. There is also a need for *in-vitro* and animal dental caries models’ study to simulate the oral cavity environment, as well as the randomized clinical trial to evaluate the anti-biofilm and anti-caries effect of Emo-Cur-NPs based aPDT.

## Data Availability

All data of this manuscript are included in the manuscript. All figures are original images and have been used for the first time in this study Any additional information required will be provided by communicating with the corresponding author via the official mail: abahador@sina.tums.ac.ir.

## Abbreviations

aPDT: Antimicrobial photodynamic therapy
BHI: Brain heart infusion
CFU: Colony forming unit
CHX: Chlorhexidine
CS: Chitosan
eDNA: Extracellular DNA
Emo: Emodin
Emo-CS-NPs: Emodin-chitosan nanoparticles
Gtf: Glycosyltransferase
ROS: Reactive oxygen species
